# Does green tea catechin enhance weight-loss effect of exercise training in overweight and obese individuals? a systematic review and meta-analysis of randomized trials

**DOI:** 10.1080/15502783.2024.2411029

**Published:** 2024-09-30

**Authors:** Farhad Gholami, Jose Antonio, Mohadeseh Iranpour, Jason Curtis, Flavia Pereira

**Affiliations:** a Shahrood University of Technology, Department of Physical Education and Sport Sciences, Faculty of Physical Education, Shahrood, Iran; b Nova Southeastern University, Department of Health and Human Performance, Davie, FL, USA; c Keiser University, Department of Exercise and Sport Science, West Palm Beach, FL, USA

**Keywords:** Catechin, obesity, exercise, weight control, lipid profile

## Abstract

**Background:**

Green tea (GT) is a common component of supplements known as fat burners. It has gained popularity as an ergogenic aid for weight reduction to assist with obesity management. This systematic review and meta-analysis aim to explore the effect of green tea ingestion coupled with exercise training (EX) on body composition and lipid profile in overweight and obese individuals.

**Methods:**

Two independent researchers systematically searched the electronic databases of PubMed, Web of Science, and Scopus. Studies with a randomized-controlled design to compare the effect of green tea in conjunction with exercise training (EX+GT) versus exercise training alone (EX+P) in overweight or obese participants were included.

**Results:**

Of the 1,015 retrieved studies, 24 were identified to undergo full-text review, out of which 10 randomized trials met the inclusion criteria. EX+GT versus EX+P had a small and consistent effect on weight [Standardized mean difference (SMD) = -0.30, CI: −0.53 to −0.07], BMI [SMD = -0.33 CI: −0.64 to −0.02] and fat reduction [SMD = -0.29, CI: −0.57 to −0.01] and there was no evidence of heterogeneity across the trials. When compared to EX+P, EX+GT had no greater effect on lipid profile improvement [triglyceride: SMD = -0.92, CI: −1.30 to 0.49; LDL: SMD = -1.44, CI: −0.73 to 0.82; HDL: SMD = 0.56, CI −0.71 to 0.46; and total cholesterol SMD = -0.54, CI −0.85 to 0.13].

**Conclusions:**

Current evidence suggests that green tea could have quite minimal additive benefit over exercise-induced weight loss. However, incorporation of green tea into exercise training does not seem to exert additional benefits on lipid profile and it warrants further investigations in the future.

## 1. Introduction

Obesity is characterized by excessive accumulation of fat resulting from an imbalance between energy intake and energy expenditure [1]. It is a prominent risk factor for numerous metabolic and non-metabolic disorders [2,3], and scientific evidence shows that a modest 5%–10% reduction in body weight elicits notable health improvements [4–6]. Lifestyle-associated behavioral interventions, including exercise training and dietary interventions, constitute pivotal components of weight management strategies aiming to mitigate obesity-associated risk factors [7,8]. Over the past few years, there has been a surge in the popularity of supplements claiming to be effective in promoting fat loss, and a growing desire among the general population seeking assistance with weight reduction efforts [9]. However, despite the inclusion of various supplements in the category of anti-obesity agents, their true weight-reducing potential remains to be determined [10,11]. Among these supplements, green tea catechins have attracted attention due to their thermogenic properties [12,13]. Green tea catechins have the potential to increase lipolysis, fat oxidation, and energy expenditure, that is claimed to assist with the management of obesity [14].

Green tea is a commonly used fat burner component proposed to enhance thermogenesis, energy expenditure, and fat oxidation [15,16]. Metabolic and fat burning potential of green tea are mainly attributed to its dominant polyphenols catechins [15]. A number of previous studies suggest that green tea has the potential to aid weight loss and alter components of lipid profile including total cholesterol and LDL cholesterol. Zhang et al. [17] indicated that habitual intake of green tea reduced body fat mass [17]. Although some studies did not find any association between green tea ingestion and obesity indices [18,19], weight reduction has been reported in some meta-analyses; for instance, Hursel et al. (2009) reported that green tea on its own, may have a small impact on weight loss by a reduction of about 1 kg [14,20]. This magnitude of the effect is too small to be clinically significant as the threshold of minimal clinically important difference in fat loss, which is defined as approximately a 2.5 kg loss [21]. Consequently, in a systematic review and meta-analysis Lin et al. (2020) proposed that incorporating green tea as a supplementary measure within weight-loss strategies that include caloric restriction and exercise training may be effective. Nevertheless, the current evidence is inconclusive, and it remains to be seen whether combining exercise training with green tea ingestion would yield greater effects than exercise training alone.

Given the widespread use of green tea catechins as prevalent adjuncts for weight reduction, it is tempting to believe that they may enhance the metabolic and weight-loss effects of exercise training. Therefore, the objective of the present systematic review and meta-analysis is to quantitively examine whether green tea ingestion in conjunction with exercise training confers a superior effect in weight loss and lipid profile improvement compared to exercise training alone in overweight and obese individuals.

## 2. Materials and methods

### 2.1. Protocol registration

The systematic review and meta-analysis were conducted under the guidelines of Cochrane Systematic Reviews of Interventions and Preferred Reporting Items for Systematic Reviews and Meta-analyses Statement (PRISMA) [22]. The registration number CRD42023437520 has been prospectively registered for the entire study protocol.

### 2.2. Literature search

A literature search by two independent reviewers was conducted using the PubMed, Scopus, and Web of Science databases for original articles from inception to November 2023. Although the weight, BMI, and fat percentage were the primary outcomes in this meta-analysis, they were not included in keywords since they might not have been directly mentioned in the title, keywords, or abstract of randomized trials. Thus, the following keywords were used in the systematic review to include the maximum number of RCTs: “green tea” OR catechin” in conjunction with “exercise” OR “training” OR “physical activity.” The entire search strategy applied in the PubMed database can be found in supplementary material 1. The search was restricted to language (English), document type (Article), and species (human) where possible. Furthermore, the reference list of all included studies were manually searched to identify studies missed in online searches.

### 2.3. Study selection

Retrieved studies were imported to EndNote X7 software (Thomson Reuters, New York, NY, USA), and duplicates were removed. Following this, screening against the predetermined inclusion and exclusion criteria was performed to define potentially eligible studies for full-text review. Two reviewers screened the studies independently, and any disagreement was resolved through discussion. The inclusion criteria were determined according to the ‘PICOS’ model (Population, Interventions, Comparisons, Outcomes, and Study Design) [23], including (i) Population: overweight or obese human subjects; (ii) Intervention: the main intervention of interest, green tea, or catechin ≥4 weeks; (iii) Comparisons: a standard or placebo treatment comparing with the intervention; (iv) Outcomes: pre- and post-test values of mean and standard deviation at both study arms for measures of body composition (weight, BMI, fat%, and fat mass) and lipid profile variables (triglyceride, LDL, HDL, and total cholesterol); and, (v) Study Design: a randomized controlled trial. The studies applying multi-ingredient supplement ingestion, non-supervised exercise training, and those with insufficient data on outcomes of interest were excluded.

### 2.4. Data extraction

Two authors independently extracted the data, and any disagreements were addressed through discussion. The following data were extracted from each study: publication data, participants’ sex, sample size, and drop-outs, exercise intervention details (i.e. modality, duration, intensity, frequency), supplementation details (dose and frequency), mean changes or mean and SD of pre and posttest values of outcome measures. If any study had more than one posttest assessment, the final data collection was included in the meta-analysis. When there was no sufficient data available, the corresponding author was contacted.

### 2.5. Quality and sensitivity appraisal

Two reviewers independently assessed the methodological quality of the included studies using the Physiotherapy Evidence Database (PEDro) scale. The PEDro scale is a valid and reliable measure of the methodological quality of randomized controlled trials in systematic reviews [24,25]. It consists of 11 items scored 0–3 poor, 4–5 fair, 6–8 good, and 9–11 excellent [26]. In addition, the leave-one-out method was performed for sensitivity analysis to define each study’s influence on overall effect size.

### 2.6. Statistical analysis

The analysis was performed using Comprehensive Meta-analysis software to assess the effect of exercise training plus supplement versus exercise training plus placebo on body composition and lipid profile in overweight and obese individuals. A random-effect model meta-analysis with a standardized mean difference was applied to compensate for the methodological diversities across included studies [27]. Change scores or mean values at baseline and post-intervention with relative standard deviations were used to calculate differences in means and 95% confidence intervals. Standard deviation was calculated from standard error or confidence intervals where required [28]. According to Cohen’s D, effect sizes <0.02 represent no effect, 0.2 to 0.5 a small effect, 0.5 to 0.8 a medium effect, and >0.8 a large effect [29]. To determine the heterogeneity of effect sizes, Higgins I^2^ statistics was used, and values were interpreted as follows: low (I^2^ ≤25%), moderate (25 < I^2^ ≤50%), high (50 < I^2^ ≤75%), and considerable (I^2^ >75%). Visual interpretation of the funnel plot and Egger’s test were used to inspect the publication bias [30]. The significance level was set at *p* < 0.05 for statistical analysis.

## 3. Results

### 3.1. Study screening and inclusion

**Figure 1.**
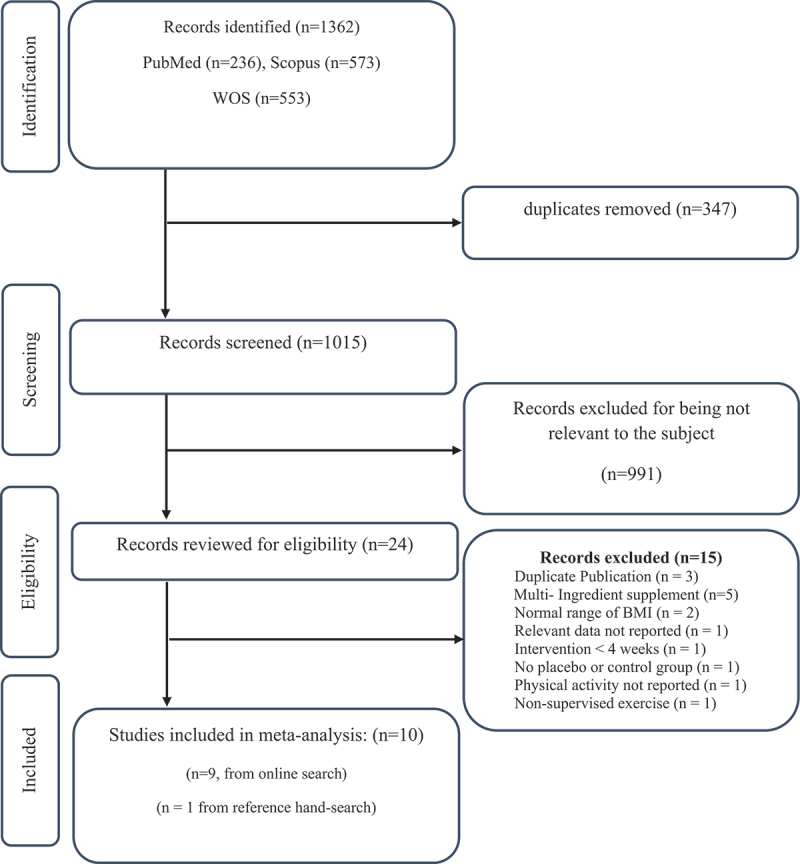
Flowchart of study selection for inclusion trials in the systematic review and meta-analysis.

The initial database search resulted in 1,362 studies, of which 347 were removed for duplication. After screening 1015 studies, 24 were found eligible for full-text review, of which 13 studies were excluded for not meeting the inclusion criteria. Of the remaining 11 studies, the study by Boroujeni et al. (2016) and Hemmatinezhad et al. (2022) were also excluded from the meta-analysis since the intervention and some other critical aspects of the research needed to be reported [31,32]. In addition to these trials, one study found by hand-search of the reference lists [33], and finally, ten studies were included in the meta-analysis. The PRISMA flow diagram provides the number of excluded studies with reasons (Figure 1).

### 3.2. Study and participants’ characteristics

An overview of the characteristics of included studies in the meta-analysis is presented in Table 1. In the included studies, the total number of participants assigned into different study arms were 476 of which 199 participants were recruited in the EX+GT arm, and 196 participants were recruited in the EX+P arm and the rest of the participants were recruited in other arms. Of 10 studies, five studies involved female participants [34–38], two studies involved male participants [39,40], and three studies involved both sexes [33,41,42]. Participants aged 21 to 70 years, with a BMI over 24.9 kg.m^−2^, were classified as overweight or obese. Regarding exercise intervention, 6 Studies employed aerobic exercise training [35–37,40–42], two studies employed high-intensity exercise training [34,39], and two studies employed concurrent exercise training [33,38]. The duration of intervention ranged from 8 weeks to 24 weeks, with 8 and 12 weeks being the most common. The frequency of exercise intervention varied from 3 to 5 d. w^−1^, most commonly with three sessions per week frequency. In all studies, participants were on a daily supplementation regimen. Green tea supplementation dosage ranged from 99 mg. d^−1^ [35] to 1500 mg. d^−1^ [34], and the most frequent supplementation dose was 500 mg. d^−1^.

**Table 1.** Study characteristics.

Author	Country	Participants	Sex	Total sample size (Drop-out)	Exercise Type	Duration	Exc. Intensity	Exc. Frequency	Supplement (dose/frequency)	Diet
Afzalpour 2017	Iran	Overweight	F	30 (0)	Anaerobic (Shuttle run training)	10 w	90% HRmax	3 d/w	Green tea (1500 mg/daily)	Habitual
Amouzadeh 2018	Iran	Overweight and Obese	F	39 (0)	Aerobic	8 w	40%-80% HRR	3 d/w	Green tea (99 mg/daily)	Habitual
Bagheri 2019	Iran	Overweight	F	31 (1)	Aerobic	8 w	40%-59% HRR	3 d/w	Green tea (500 mg/daily)	Habitual
Gahreman 2016		Overweight	M	48 (5)	Interval Spring	12 w	85-90% HRmax	3 d/w	Camellia sinensis extract (750 mg/daily)	Habitual
Hill 2007	Australia	Overweight and Obese (Postmenopausal women)	F	42 (4)	Aerobic	12 w	75% HRmax	3 d/w	Green tea (300 mg EGCG/daily)	Habitual
Hosseini 2021	Iran	Overweight	F	40 (unknown)	Concurrent (resistance and aerobic)	8 w	61-88% HRmax 40-75% 1RM	3 d/w	Green tea (500 mg GT containing 300 mg C/daily)	Habitual
Maki 2009	US	Overweight and Obese	M & F	137 (30)	Aerobic	12 w	Moderate intensity	≥3 d/w	Green tea (500 mg GT beverage 625 mg C/daily)	Habitual
Roberts2021	UK	Overweight	M & F	31 (4)	Aerobic	8 w	75-80% HRmax	3-5 d/w	Decaffeinated green tea extract (580 mg dGTE containing 400 mg EGCG/daily)	Habitual
Zhang 2020	China	Overweight and Obese	M	24 (0)	Walking	12 w	50-80% HRmax	4 d/w	Grean tea extract (300 mg EGCG/daily)	Habitual
Belcaro 2013	Italy	Metabolic Syndrome	M & F	100 (2)	Concurrent (resistance and aerobic)	24 w	Moderate intensity	Unknown	Greenselect Phytosome (300 mg/daily)	Calorie deficit (750-1000 Kcal)

Exc: Exercise, HRR: Heart Rate Reserve, 1RM: One Repetition-Maximum, HRmax: Maximum Heart Rate, F: Female, M: Male, RPE: rate of Perceived Excretion, EGCG, Epigallocatechin Gallate.

### 3.3. Meta-analysis

#### 3.3.1. Body composition measures

**Figure 2.**
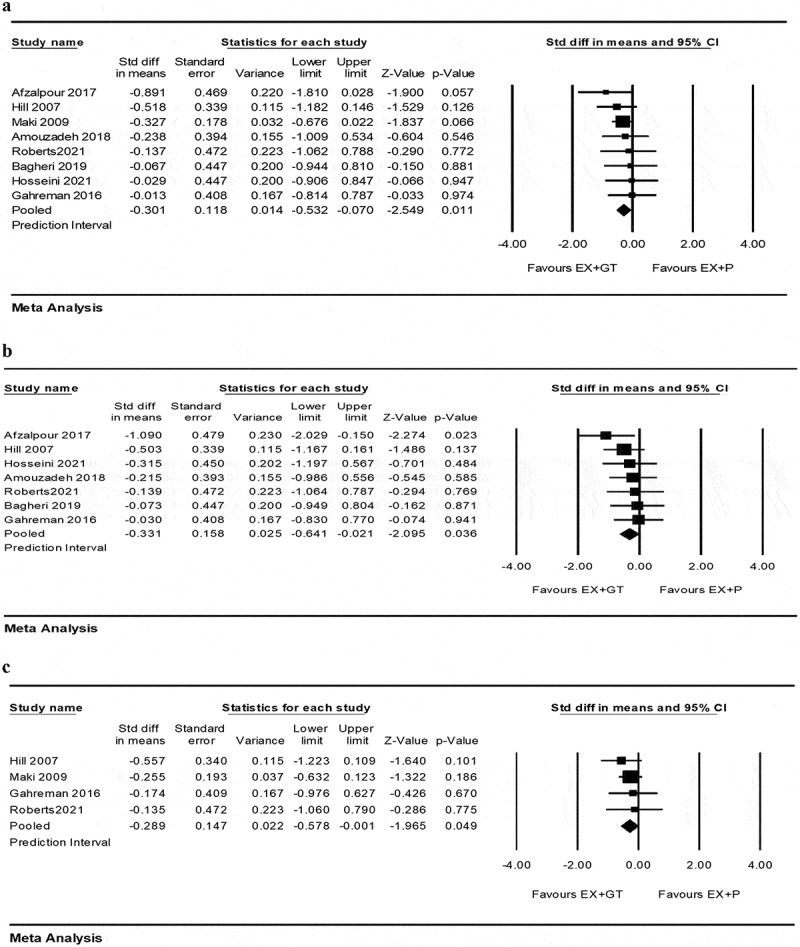
Forest plot of the effect of EX+GT versus EX+P on body composition. Data are presented as SMD with 95% confidence intervals. (a) weight, (b) BMI and (c) fat mass.

Meta-analysis of variables revealed that the study by Belcaro et al. (2013) was an outlier that was removed from the analysis due to the exaggerated effect size, distorting the overall result and increasing the heterogeneity [33]. Eight studies were included [34–39,41,42] indicating that EX+GT had significantly greater effect on weight reduction compared to EX+P [SMD = −0.30 (95% CI −0.53 to −0.07), *p* = 0.011; 8 trials]. There was no evidence of heterogeneity across the trials (I^2^ = 0.000, *p* = 0.85). In addition, a meta-analysis of 7 studies [34–39,42] regarding BMI indicated that compared to E+P, EX+GT had significantly greater effect on BMI reduction [SMD = −0.33 (95% CI −0.64 to −0.02), *p* = 0.03; 7 trials] with no evidence of heterogeneity across the trials (I^2^ = 0.000, *p* = 0.69). Four studies included in the meta-analysis of fat mass [37,39,41,42] indicating that EX+GT had significantly greater effect on fat reduction compared to EX+P [SMD = −0.29 (95% CI −0.57 to −0.01), *p* = 0.049; 4 trials]. There was no evidence of heterogeneity across the trials (I^2^ = 0.000, *p* = 0.84) (Figure 2).

EX+GT:exercise training plus green tea; EX+P: exercise training plus placebo

#### 3.3.2. Lipid profile

**Figure 3.**
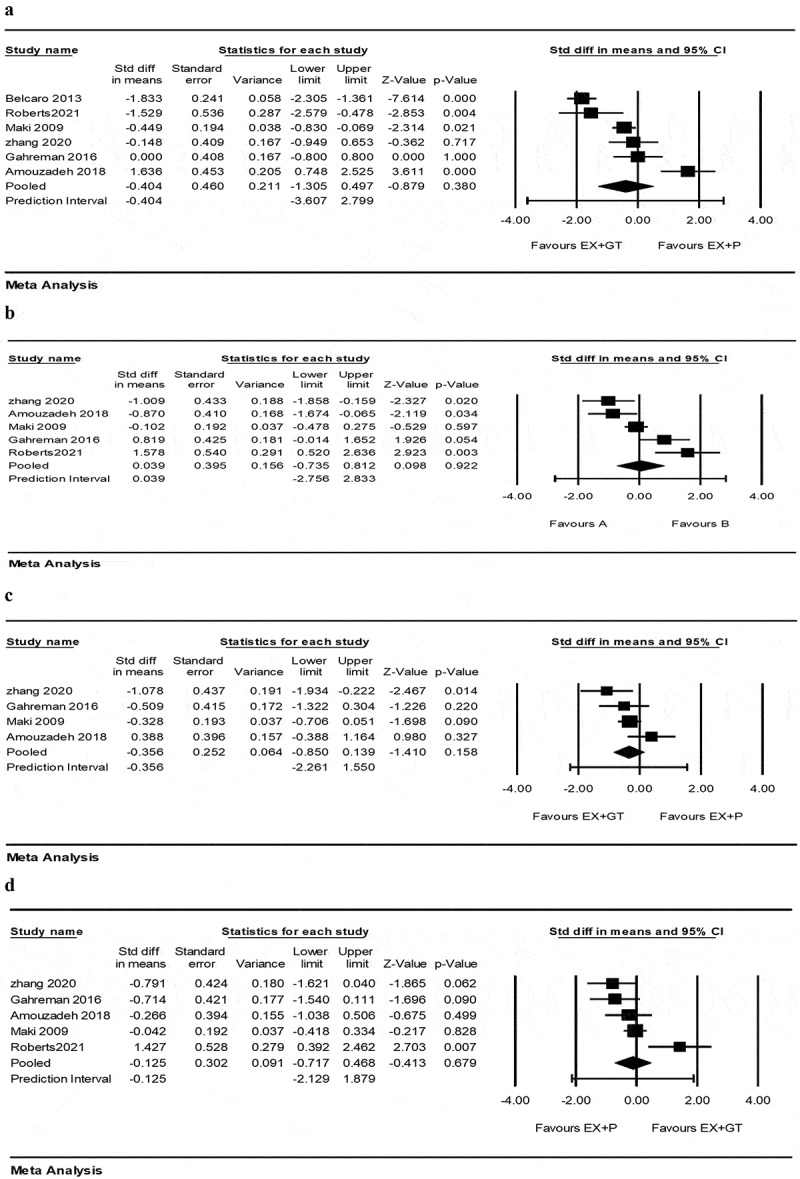
Forest plot of the effect of EX+GT versus EX+P on lipid profile. Data are presented as SMD with 95% confidence intervals. (a) triglyceride, (b) LDL, (c) total cholesterol and (d) HDL.

Meta-analysis of 6 studies [33,35,39–42] on triglyceride indicated that EX+GT had no significant effect on TG reduction compared to EX+P[SMD = −0.40 (95% CI −1.30 to 0.49), *p* = 0.38; 6 trials]. There was also a significantly considerable heterogeneity across the trials (I^2^ = 91.29, *p* = 0.0001). Five studies [35,39–42] were included in the meta-analysis of LDL, indicating that EX+GT had no significantly greater effect on LDL reduction compared to EX+P [SMD = 0.03 (95% CI −0.73 to 0.82), *p* = 0.92; 5 trials]. The heterogeneity of the trials was also significantly considerable (I^2^ = 81.98, *p* = 0.0001). Six studies [35,39–42] were included for HDL, showing that EX+GT had no greater effect on HDL compared to E+P, [SMD = −0.12 (95% CI −0.71 to 0.46), *p* = 0.67; 6 trials]. There was also a significantly high heterogeneity across the trials (I^2^ = 70.10, *p* = 0.01). Four studies [35,39–41] were also included for total cholesterol and indicated that EX+GT had no greater effect on total cholesterol reduction compared to EX+P [SMD = −0.35 (95% CI −0.85 to 0.13), *p* = 0.15; 4 trials]. The heterogeneity of the included trials was high but insignificant (I^2^ = 53.15, *p* = 0.09) (Figure 3).

EX+GT:exercise training plus green tea; EX+P: exercise training plus placebo

#### 3.3.3. Quality and publication bias

The quality assessment of the included studies was performed using the PEDro tool. The quality of the included studies was fair to excellent, with the score ranging from 5 [34,38] to 9 [40] and an average score of 6.7, summarized in supplementary material 2. The attrition rate was low, with an average of 9% drop-out. Visual inspection of funnel plots and Egger’s test regarding green tea studies did not identify publication bias for weight (*p* = 0.70), BMI (*p* = 0.66), fat mass (*p* = 0.27), triglyceride (*p* = 0.90), total cholesterol (*p* = 0.15), and HDL (*p* = 0.46).

## 4. Discussion

The current systematic review and meta-analysis aimed to answer for the first time whether green tea catechin coupled with exercise training can further enhance the exercise-induced improvement of weight and lipid profile in overweight and obese individuals. Overall, results indicated that green tea supplementation alongside exercise training was effective in reducing weight-control measures with a small and consistent effect. However, green tea consumption coupled with exercise training had no additional effect on lipid profile, including triglyceride, LDL, HDL, and total cholesterol.

Previous studies indicate that green tea elevates energy expenditure and induces fat oxidation rate upon ingestion. Bérubé-Parent et al. (2005) conducted a study and demonstrated that the ingestion of green tea catechin with caffeine resulted in a daily increase in energy expenditure of 750 kJ [15]. Hursel et al. (2011) further supported the thermogenic effect of these substances. They found that green tea catechin led to increased daily energy expenditure, and the effect was greater when combined with caffeine [16]. These findings have prompted individuals struggling with obesity and clinicians to incorporate green tea catechin into their weight-loss strategies; thus, it is now considered an integral component of fat burner supplements [43]. As a result, the utilization of green tea catechin has become increasingly prevalent in the belief that it can provide further assistance with obesity management alongside dietary interventions and exercise training.

The primary goal of exercise and dietary interventions in obese individuals is to enhance overall health and well-being, besides reducing fat mass. Obesity is recognized as the principal etiological factor for metabolic dysfunctions and the elevation of blood triglyceride levels, total cholesterol, and low-density lipoprotein (LDL). Conversely, green tea consumption has been documented to elicit cardiometabolic effects [14,44,45]. Given catechins’ cardiometabolic and thermogenic potential, it is plausible to postulate that using them in conjunction with exercise training may yield greater weight loss outcomes.

A comprehensive review and meta-analysis examining the pooled effect of green tea intake along with exercise training on weight reduction and lipid profile are currently lacking. However, some previous meta-analyses investigated the effects of green tea against placebo on cardiometabolic measures and weight reduction. Hursel et al. (2009) reported a significant reduction in weight due to green tea ingestion [20]. A recent meta-analysis by Lin et al. (2020) also indicated that green tea ingestion is likely effective in reducing weight and BMI in obese individuals [14]. They also suggested that the magnitude of the effect is dose-dependent, with a greater prominence observed in treatment duration longer than 12 weeks and dosage of <500 mg.d^−1^ [14]. Indeed, the effect sizes reported by the meta-analysis above seemed to be small. As such, Lin et al. (2020) suggested that green tea ingestion combined with a balanced diet and exercise training may be more effective in the management of obesity. In line with this study, we observed a small effect size for weight reduction by green tea consumption coupled with exercise training.

Our findings also indicate that the incorporation of green tea into exercise training yielded no greater effect on lipid profile measures compared to exercise training alone. This is comparable and, to some extent, in line with previous meta-analysis comparing green tea only versus placebo on lipid profile [45,46]. In a meta-analysis by Onakpoya et al. (2014), a small reduction of total cholesterol and LDL was reported by green tea ingestion versus placebo, yet, no significant effect was defined for triglyceride and HDL [45]. Asbaghi et al. (2020) also reported a small reduction of triglyceride by green tea versus placebo. They also found no significant effect of green tea on LDL and HDL in type-2 diabetic patients [46]. The lipid lowering effect of green tea is attributed to its active components named catechins [47,48]. Catechins increase thermogenesis and also interfere with the emulsification, digestion, and micellar solubility [49,50]; thus, suggested as effective and safe lipid-lowering agents [50]. Exercise training is also well-known to improve lipid profile in obese individuals. Thus, one might assume that green tea and exercise training might have a synergistic effect when implemented in conjunction; however, current scientific evidence does not support this belief and it needs to be further investigated in future studies.

## 5. Limitations

The study includes some strengths and limitations to be acknowledged. This is the first meta-analysis to assess the effect of green tea coupled with exercise training on weight measures and lipid profile in overweight and obese individuals. It is also important to highlight that this meta-analysis expressly excluded studies involving multi-ingredient supplements to isolate the effects solely attributable to green tea. Despite an extensive online search yielding 1362 studies, only ten studies were deemed suitable for inclusion. This shows that despite assertions regarding the efficacy of green tea as a complimentary weight-loss approach alongside exercise training, the available evidence needs to be more sufficient to draw definitive and consistent conclusions, thereby warranting further research. Notably, a considerable degree of heterogeneity was observed in certain variables, which can be attributed to the limited number of studies included. The inclusion of English-language studies may introduce the possibility of publication bias; however, the exclusion of non-English articles usually does not have a significant effect on the results of systematic reviews [51].

## 6. Conclusion

Based on the findings of the present meta-analysis, green tea catechin intake along with the exercise training has quite minimal but consistent effect on weight reduction when compared to exercise training alone. In addition, based on a handful of current evidence, incorporation of green tea into exercise training has no additional effect on lipid profile; additionally, the substantial heterogeneity observed calls for more rigorous well-designed prospective studies.

## Data Availability

The relevant analysis of data has been presented in the manuscript or supplementary materials. The datasets generated during and/or analyzed during the current study are also available from the corresponding author on request.
